# ZIP code-level alcohol outlet density and nonfatal overdose among people who inject drugs in 22 US metropolitan statistical areas: a multilevel modeling analysis

**DOI:** 10.1186/s12954-026-01414-0

**Published:** 2026-03-02

**Authors:** Snigdha R. Peddireddy, Stephanie Beane, Courtney Yarbrough, Umedjon Ibragimov, Janet R. Cummings, Danielle F. Haley, Sabriya L. Linton, Hannah L.F. Cooper

**Affiliations:** 1 Department of Behavioral, Social, and Health Education Sciences, Rollins School of Public Health, Emory University, 1518 Clifton Road NE, Atlanta, GA 30322 USA; 2 Department of Health Policy and Management, Rollins School of Public Health, Emory University, 1518 Clifton Road NE, Atlanta, GA 30322 USA; 3 Department of Community Health Sciences, School of Public Health, Boston University, 715 Albany Street, Boston, MA 02118 USA; 4 Department of Mental Health, Bloomberg School of Public Health, Johns Hopkins University, 615 N Wolfe Street, Baltimore, MD 21205 USA; 5 Center of Population Sciences for Health Empowerment, College of Nursing, Florida State University, 98 University Way, Tallahassee, FL 32306 USA

**Keywords:** Alcohol outlet density, Overdose, Opioids, Equity, SDOH

## Abstract

**Background:**

Alcohol outlet density (AOD) is associated with drinking behaviors and related harms across several populations. As alcohol consumption compounds the depressive effects of opioids to increase overdose risk among people who inject drugs (PWID), this study investigated (1) whether off-premise AOD is associated with the risk of nonfatal overdose among a large sample of PWID, and (2) whether this relationship varies by individual race/ethnicity.

**Methods:**

We linked individual-level 2018 National HIV Behavioral Surveillance (NHBS) data with ZIP code-level data on off-premise AOD in 2016 from the US Census Bureau’s ZIP Code Business Pattern survey. NHBS surveys PWID across 22 metropolitan statistical areas (i.e., urban cores and surrounding counties with ≥ 50,000 residents, defined by commuting patterns) using respondent-driven sampling. Hierarchical generalized linear models quantified the association between AOD and nonfatal overdose, overall and by PWID race/ethnicity.

**Results:**

The sample comprised 9,764 PWID who used opioids, 45% of whom were non-Hispanic/Latine White, 38% non-Hispanic/Latine Black, and 18% Hispanic/Latine. Over a quarter (28%) experienced an overdose in the past year. The median outlet density was 4.0 outlets per square mile. The adjusted model did not show a significant relationship between AOD and odds of overdose (OR: 1.01, 95% CI 1.00–1.02), and the relationship remained null in the race/ethnicity interaction model.

**Conclusions:**

Regulating AOD alone may not effectively mitigate overdose risk among PWID. Future research should explore why overdoses among PWID are not sensitive to changes in AOD. Possible explanations to consider are whether (1) AOD is unassociated with alcohol consumption among PWID, or (2) PWID stagger the timing of their alcohol consumption so it does not coincide with opioid consumption.

## Introduction

Provisional drug overdose data from the United States (US) indicate that there were approximately 55,000 opioid-involved overdose deaths in 2024 [1]. While most recent research has focused on stimulant co-involvement in the fourth and latest wave of the opioid epidemic [2], alcohol has also played a significant role in opioid overdoses: alcohol was involved in approximately 17% of opioid overdose deaths in the US in 2020, up 41% from 2019 [3], with more recent reports from Oregon (2021) and Vermont (2023) indicating alcohol involvement in nearly 20% of opioid overdose deaths [4, 5]. The concurrent consumption of alcohol and opioids has synergistic effects that amplify the respiratory depressant properties of both substances, and significantly elevate the risk of overdose [6, 7].

Evidence suggests that alcohol consumption in the general population is influenced, in part, by the density of off-premise alcohol outlets (i.e., outlets where alcohol is sold for consumption off retail outlet property, such as grocery and convenience stores). Off-premise alcohol outlet density (AOD) has consistently been linked to harmful drinking behaviors and related acute harms [8–11]. This body of evidence has supported the regulation of AOD as a means to mitigate these risks in the general population [11–13]. AOD regulation typically involves zoning and licensing policies, such as limiting areas where alcohol sales are allowed or setting limits on the number of licensed outlets according to set criteria, respectively, enabling local authorities to reshape neighborhood alcohol risk environments [14].

Despite the established connection between AOD and alcohol consumption in the general population, as well as between alcohol and opioid co-use and overdoses among people who use drugs, exploration into AOD’s relationship with drug overdose has been limited. To date, only two studies have investigated this relationship: Nesoff et al. found that, in Baltimore city, each additional off-premise outlet in a census block group was associated with a 16.6% increase in the rate of overdoses from any drugs among the general population of residents [15]. Similarly, Viera et al. found that increased off-premise AOD at the census tract level in Connecticut was associated with a 13% increase in the proportion of alcohol-involved opioid overdose deaths specifically [16]. These studies provide an important foundation for understanding how alcohol environments shape overdose risk; however, it remains uncertain how these findings extend specifically to people who inject drugs (PWID). PWID face a heightened risk of overdose compared to people who consume opioids through other administration routes, as injecting introduces drugs into the bloodstream more immediately and with greater potency [17]. Thus, it is important to assess whether associations similar to those observed in prior studies emerge when examining overdose risk among PWID, a population for whom patterns of substance use, harm reduction engagement, and environmental exposures may differ substantially from those of the general population.

Additionally, aggregate data may obscure differential impacts on marginalized subgroups. Specifically, the same level of exposure to alcohol outlets may have more deleterious impacts on racially minoritized individuals. Between 2017 and 2020 in Illinois, the prevalence of alcohol co-involvement in opioid overdose deaths was 39% and 33% among Hispanic/Latine and non-Hispanic/Latine Black decedents, respectively, compared to 27% among White decedents [18]. Further, racially minoritized groups may disproportionately cluster in neighborhoods with high AOD [19–24], intensifying already growing opioid overdose rates among these groups [25–28]. This study thus aimed to explore whether (1) off-premise AOD is associated with the risk of nonfatal overdose among a large sample of PWID and (2) the relationship between AOD and overdose varied by individual race/ethnicity.

## Methods

### Overview

This analysis utilized data from the Centers for Disease Control and Prevention’s (CDC) National HIV Behavioral Surveillance (NHBS) study, the largest and most racially/ethnically diverse source of health data from PWID across multiple metropolitan statistical areas (MSAs) in the U.S. MSAs span every U.S. geographic region and are urban cities and surrounding counties with a population of at least 50,000 connected by commuting patterns. The CDC implemented NHBS to monitor behavioral risk and protective factors associated with HIV. In 2018, NHBS aimed to gather data from approximately 500 PWID in each of the 23 MSAs using respondent-driven sampling (RDS).

This multilevel cross-sectional study linked 2018 NHBS data on 9,764 individual PWID with ZIP code-level AOD data from the US Census Bureau’s 2016 ZIP Code Business Pattern (CBP) survey. We used 2016 CBP data because complete AOD data were no longer available after this year due to the CBP survey’s suppression of establishments in ZIP codes with fewer than three outlets, preventing the construction of comparable measures for later years. The resulting two-year lag between exposure and outcome is consistent with prior work and may allow time for alcohol risk environments to influence overdose risk through structural and behavioral pathways [29, 30]. To assess the stability of AOD in a two-year period, we compared distributions of AOD from 2014 to 2016: the Spearman correlation was 0.97, indicating a highly stable retail environment over this period, further supporting the appropriateness of a two-year lag.

### Sample

PWID were recruited to the NHBS study if they (1) reported injecting a non-prescribed drug in the past 12 months, (2) were aged 18 years or older, (3) lived in a participating MSA, (4) had not previously participated in the current cycle of data collection, and (5) could complete the survey in English or Spanish. This analysis further restricted the sample to participants who reported past-year injection or non-injection use of a non-prescribed opioid (99% of the initial sample). The analytic sample excluded (1) participants who reported a race/ethnicity other than non-Hispanic/Latine White, non-Hispanic/Latine Black, or Hispanic/Latine due to sparse data for other races/ethnicities and (2) participants with missing values for variables used in this analysis (< 5% of the sample). Participants from the San Juan-Bayamon MSA were excluded as well because of a lack of racial/ethnic diversity, resulting in 22 MSAs represented in the analytic sample.

### Measures

#### Outcome

*Past 12-month non-fatal opioid overdose.* The outcome was a dichotomous variable indicating whether the NHBS participant experienced a non-fatal opioid overdose in the past 12 months based on the following question: “In the past 12 months, did you overdose on heroin or painkillers? By overdose, I mean if you passed out, turned blue, or stopped breathing from using drugs.”

#### Primary independent variable

*ZIP code-level alcohol outlet density.* The primary independent variable was the ZIP code-level density of off-premise alcohol outlets, calculated as the number of outlets divided by the square miles in each Zip Code Tabulation Area (ZCTA), defined as the US Postal Service’s ZIP code service areas [31]. Data on the numerator were obtained from the 2016 U.S. Census Bureau’s ZIP Code Business Pattern (CBP) data [32]; the two-year lag allowed for delayed impacts on the outcome. The CBP reports annual, sub-national economic data on business establishments by industry type. Because the CBP does not explicitly identify alcohol outlets, we used North American Industry Classification System (NAICS) codes to capture establishment types that may sell alcohol off-premise, as outlined by Matthews et al. [33]. To address state-level variation in retail alcohol licensing, we consulted state liquor control and licensing boards to determine which of these NAICS-coded establishments were legally permitted to sell alcohol during the study period; only eligible establishment types were counted as outlets. ZIP codes were matched to ZCTAs using HUD-USPS ZIP Crosswalk files. PWID were linked to 2016 AOD using the reported ZIP code of residence.

#### Covariates

*State-level covariates Alcohol policy scores* quantified the strengths of states’ alcohol policy landscapes in 2017 [34]. Scores ranged from 0 to 100 and higher scores represented more comprehensive and restrictive alcohol environments. Indicators included policies regarding alcohol production and distribution, sales, and consumption. PWID were linked to states using the reported county of residence.

*ZIP code-level covariates* We used 2017 (one-year lag) United States Postal Services Address Information Systems data [35] to construct *(1) residential and (2) business vacancies* by dividing the monthly average of vacancies in each category by ZCTA square miles. Residential and business vacancies shape excessive drinking and drug-related behaviors, as well as opioid-related overdoses [36, 37]. We also calculated 2017 (one-year lag) *percent of people with income at or below the federal poverty line* using the midpoint of the U.S. Census Bureau’s American Community Survey 5-year block data [38]. We did not include a measure of racial segregation in our models due to potential multicollinearity with AOD, as indicated by the racialized concentration of alcohol outlets [19–24].

*Individual-level covariates* Variables capturing individual-level characteristics were drawn from NHBS. These included participant sociodemographic characteristics (race/ethnicity, sex, age, education, employment), poverty status per the U.S. DHHS Poverty Guidelines [39], health insurance status, daily injection drug use, incarceration (past 12 months), unhoused status (past 12 months), HIV serostatus, self-reported disability status using the DHHS data standard items [40, 41], psychological distress as measured by the Kessler-6 scale [42], network size, respondent status as an RDS seed, and receipt of sterile injection drug use equipment from a syringe services program (SSP) in the past year. Although NHBS does not directly measure receipt of naloxone or overdose education, SSPs often offer these other services and, therefore, their utilization serves as a limited proxy for engagement in overdose prevention supports [43, 44].

#### Potential effect modifier

*Individual race/ethnicity.* NHBS participants’ self-reported data informed three mutually exclusive racial/ethnic groups: non-Hispanic/Latine White (hereinafter ‘White’), non-Hispanic/Latine Black (hereinafter ‘Black’), and Hispanic/Latine. When participants reported that they belonged to two racial groups and were not Hispanic/Latine, this study followed the Office of Management and Budget’s “plurality” guidelines to assign them to a single racial/ethnic group using the federal Office of Management and Budget’s “plurality” guidelines [45].

### Analysis

Statistical analyses were carried out with SAS version 9.4. We first explored distributions of all variables, examined correlations, and checked for multicollinearity. Age and number of years since first injection were highly correlated (0.75), so injection duration was removed from all models. We conducted a cross-tabulation and chi-square test to discern statistically significant differences in past 30-day binge drinking among individuals who either experienced or did not experience an overdose in the past year. We also compared median alcohol outlet densities by individual PWID race/ethnicity using a Kruskal-Wallis test [46].

Three-level hierarchical generalized linear models with a logit link [47] examined the associations between ZIP code-level AOD and nonfatal opioid overdose. Models included random intercepts for PWID clustered within ZIP codes and ZIP codes clustered within MSAs. Models could not support a random intercept for both MSA and state, as only three states contained more than one MSA (unconditional models with MSAs and states resulted in a state-level variance component of zero [*p* > 0.05] and a warning that the estimated G matrix was not positive definite). Previous work has shown that when the highest level (here, state) of clustering is ignored in a model, that level’s variance component is redistributed to the lower level [48], so models only included a random intercept for MSA as the highest spatial level of clustering.

Three models are reported: (1) an unadjusted model, (2) a multivariable model adjusted for state, ZIP code, and individual PWID covariates and (3) an adjusted multivariable model with interactions to explore whether the relationship of AOD to nonfatal opioid overdose varied by race/ethnicity. We express model outcomes as odds ratios for overdose where the AOD is set to increments of three outlets per square mile above the median AOD (equivalent to about half of the interquartile range). This approach facilitates comprehension of the relationship between a higher AOD and the odds of nonfatal overdose. Additionally, we based AOD increments on the median, as the distribution of AODs was highly skewed in our sample. While we report the prevalence of binge drinking in our sample for descriptive purposes, this measure was not included in the models as it is a potential mediator in the relationship between AOD and overdose.

## Results

### Sample description

The sample of 9,764 PWID lived in 20 states and 22 MSAs. Approximately two-thirds (68%) of the sample were male; 45% were White, 38% were Black, and 18% were Hispanic/Latine (Table 1). The majority of participants exhibited indicators of socioeconomic hardship: approximately 75% subsisted at or below the federal poverty guidelines, 59% were unemployed, and 25% were unable to work due to poor health. Regarding health indicators, 89% reported daily injection drug use, 39% experienced psychological distress in the past month, and 68% had a qualifying disability. Importantly, 28% of participants reported experiencing an opioid overdose in the past year. The prevalence of binge drinking among our sample (27%) was similarly high. Among PWID who had overdosed in the past year, 31% reported binge drinking in the past month, and, among those who did not overdose, 26% reported binge drinking. This difference was statistically significant (χ2 = 26.4, *p* < 0.0001).

**Table 1:** ZIP, state, and participant characteristics, overall and by opioid overdose, 2018 Centers for Disease Control and Prevention’s National HIV Behavioral Surveillance (NHBS) data (*n* = 9,764)

Characteristic	Overall (*n* = 9,764)	Opioid overdose = No (*n* = 6,992; 72%)	Opioid overdose = Yes (*n* = 2,772; 28%)
	Mean (SD) or *N* (%)		
ZIP (*n* = 1,209 unique ZIP codes)			
Alcohol outlet density, mean (SD)	12 (25)	–	–
Business vacancy density, mean (SD)	64 (326)	–	–
Residential vacancy density, mean (SD)	54 (116)	–	–
Percent of residents with income *≤* federal poverty level^a^, mean (SD)	17 (10)	–	–
*State (n = 20)*			
Alcohol policy score, mean (SD)	38 (9)	–	–
*Individual (NHBS)*			
Age, mean (SD)	44 (13)	45 (13)	41 (12)
Race/ethnicity^b^			
Non-Hispanic/Latine Black	3,684 (38)	2,892 (41)	792 (29)
Hispanic/Latine	1,726 (18)	1,200 (17)	526 (19)
Non-Hispanic/Latine White	4,354 (45)	2,900 (41)	1,454 (52)
*Sex* ^b^			
Male	6,667 (68)	4,787 (68)	1,880 (68)
Female	3,016 (31)	2,145 (31)	871 (31)
Missing	81 (1)	60 (1)	21 (1)
Income at or below the federal poverty level	7,288 (75)	5,248 (75)	2,040 (74)
High school diploma or equivalent	7,015 (72)	5,028 (72)	1,987 (72)
*Employment* ^b^			
Not employed/other	5,806 (59)	4,017 (57)	1,789 (65)
Employed full or part-time	1,476 (15)	1,124 (16)	352 (13)
Unable to work due to health	2,482 (25)	1,851 (26)	631 (23)
Daily injection	8,654 (89)	6,058 (87)	2,596 (94)
HIV positive	585 (6)	430 (6)	155 (6)
Incarceration (past 12-months)	3,602 (37)	2,230 (32)	1,372 (49)
Unhoused (past 12-months)	6,588 (67)	4,384 (63)	2,204 (80)
Insured	7,315 (75)	5,244 (75)	2,071 (75)
Received clean needles from an SSP^c^	5,141 (53)	3,421 (49)	1,720 (62)
Psychological distress (past 30 days)	3,782 (39)	2,403 (34)	1,379 (50)
Disability	6,603 (68)	4,637 (66)	1,966 (71)

^a^Data derived from the Census Bureau - American Community Survey, not individual NHBS data

^b^Percentages may not add to 100% due to rounding

^c^Syringe service program

Among the 1,209 unique ZIPs where NHBS PWID resided, the mean AOD was 12 (SD = 25) off-premise alcohol outlets per square mile, and the median was 4.0 (range: 0–266.16); the distribution of AODs among our sample was positively skewed.

### Model-based analysis

In the unadjusted model, as ZIP code-level AOD increased by three outlets per square mile above the median AOD (4.0), the odds of overdose among PWID in our sample increased by 2% (OR: 1.02; 95% CI 1.01–1.03, *p* = 0.003) (Table 2). However, the magnitude of this relationship was attenuated to non-significance (OR: 1.01, 95% CI 1.00–1.02, *p* = 0.23) in the adjusted model controlling for state-level alcohol policy scores and ZIP code- and individual-level covariates. In the race/ethnicity interaction model, interaction terms for AOD*Black or AOD*Hispanic/Latine, compared to the White reference group, were not statistically significant (*p* = 0.16–0.17).

**Table 2:** Unadjusted and adjusted hierarchical generalized linear regressions^a^ of the odds of non-fatal opioid overdose on alcohol outlet density and race/ethnicity potential effect modifier, 2018 Centers for Disease Control and Prevention’s National HIV Behavioral Surveillance (*N* = 9,764)

Unadjusted model	OR (95% CI), *p* value
Alcohol outlet density^b^	1.02 (1.01, 1.03), 0.003
Var (SE) for MSA	0.15 (0.05), 0.002
Var (SE) for ZIP	0.05 (0.02), 0.006
*Adjusted* ^c^ *model*	
Alcohol outlet density^b^	1.01 (1.00, 1.02), 0.23
Race/ethnicity (Ref: Non-Hispanic/Latine White)	
Non-Hispanic/Latine Black	0.80 (0.70, 0.91), < 0.001
Hispanic/Latine	0.96 (0.83, 1.10), 0.53
Var (SE), p value for MSA	0.11 (0.04), 0.004
Var (SE), p value for ZIP	0.007 (0.01), 0.30
*Adjusted model* ^c^ *including interaction with race/ethnicity*	
Alcohol outlet density^b^	1.01 (1.00, 1.03), 0.06
*Race/ethnicity (Ref: Non-Hispanic/Latine White)*	
Non-Hispanic/Latine Black	0.83 (0.71, 0.95), 0.009
Hispanic/Latine	1.00 (0.85, 1.16), 0.97
Alcohol outlet density * race/ethnicity, ORR^d^	
Non-Hispanic/Latine Black	0.99 (0.99, 1.002), 0.17
Hispanic/Latine	1.00 (0.99, 1.001), 0.16
Var (SE),* p* value for MSA	0.10 (0.04), 0.004
Var (SE),* p* value for ZIP	0.007 (0.01), 0.30

^a^3-level (Level 1: Individual PWID, Level 2: ZIP, Level 3: MSA) hierarchical generalized linear model with logit link

^b^OR offset 3 (approximately half the interquartile range) from the median alcohol outlet density for unique ZIP codes (4.04)

^c^Model adjusted for state-level alcohol policy score, ZIP-level covariates (density of business and residential vacancies, percent of residents with income at or below the federal poverty level), and individual covariates (race/ethnicity, sex, age, income, education, employment, health insurance, incarceration, daily injection status, unhoused status, HIV status, network size, receipt of clean needles from an SSP, psychological distress, and disability status)

^d^Odds ratio ratio

As a sensitivity analysis, we ran models excluding alcohol policy scores. The relationship between AOD and overdose was still not statistically significant in sensitivity analysis models (no or negligible change to estimates and confidence intervals).

## Discussion

Leveraging the CDC’s comprehensive NHBS data allowed this analysis to generate three important findings. First, a high prevalence of nonfatal opioid overdose was observed among PWID—with more than one in four participants reporting an overdose in the past year—and a higher percentage of those who had overdosed reported binge drinking compared to those who had not overdosed. Second, while AOD and overdose were significantly associated in the unadjusted model, this relationship did not persist after adjusting for potential confounders. Lastly, the relationship between AOD and overdose did not vary by race/ethnicity.

The prevalence of binge drinking in our sample of PWID is notably higher than the 22% reported for the general U.S. adult population [49] and aligns with more localized studies documenting similarly high rates (19–40%) among PWID [50–55]. The higher proportion of binge drinking among PWID who had overdosed highlights the significance of polysubstance use in shaping overdose vulnerability. As injecting opioids already confers heightened overdose risk due to its rapid and potent pharmacologic effects [17], concurrent excessive alcohol use may exacerbate this risk through additive depressant effects on respiration. Incorporating tailored strategies in harm reduction services, such as electronic screening and brief intervention for excessive alcohol use [56] and targeted naloxone distribution for PWID who report binge drinking [57], may therefore represent critical avenues for mitigating overdose risk among PWID.

In contrast to prior research in Baltimore city [15] and Connecticut [16] that found a positive association between off-premise AOD and overdose at the census block group and census tract levels, respectively, we did not identify a significant relationship between AOD and self-reported nonfatal overdose among PWID after adjusting for confounders. A related ecological study in New Hampshire used alcohol sales volume rather than AOD as a proxy for availability and found that on-premise alcohol sales were positively associated with opioid overdose mortality at the ZIP code level, while off-premise sales were inversely associated [58]. Several reasons may explain these disparate findings. We analyzed this relationship among PWID specifically, rather than the general population, and PWID may carefully manage the timing of their alcohol consumption in relationship to their opioid consumption. Insights from a qualitative study of PWID lend credence to this possibility: Edsall et al. found that PWID avoid alcohol while using heroin to (1) mitigate riskier heroin use that could follow alcohol consumption and thus lead to an increased risk of overdose, and (2) to avoid alcohol’s blunting of opioid’s effects [59]. PWID may be more knowledgeable about overdose prevention—including about alcohol’s amplification of opioid overdose risk—than people who primarily use drugs through other administration routes [60, 61]. We could not examine these potential confounders. Another potential explanation for the lack of a significant relationship between AOD and nonfatal overdose in our study, as opposed to city- and state-specific findings [15, 16, 58], might be the nationwide scope of our analysis. Investigating 22 MSAs as opposed to a single city, state, or region could introduce heterogeneity that obscures any distinct, localized associations. Additionally, our study necessarily focused on nonfatal overdoses, because overdose was ascertained via self-report, while other studies included or only focused on fatal overdoses [15, 16]. Possibly, AOD is associated with higher rates of fatal overdose because of alcohol’s amplifying effects on respiratory depression. To extend these lines of inquiry, future research may incorporate more granular and multidimensional measures of alcohol access—including physical availability, access, and consumption patterns—and distinguish between opioid-only overdoses and alcohol-involved opioid overdoses, ideally linking ecological indicators with individual-level behavioral data to more precisely isolate the relationship between alcohol environments and overdose risk among PWID.

No prior studies have examined whether alcohol availability differentially affects overdose risk among PWID—or among people who use opioids more broadly; our findings contribute preliminary evidence that this relationship may be null across the racial/ethnic groups included in this study. In the general population, stronger AOD regulations have been linked to lower odds of binge drinking among White but not Black or Hispanic/Latine residents [9]. These findings suggest that, although alcohol availability may influence drinking behaviors differently across racial/ethnic groups, such patterns do not appear to translate into differential overdose risk among PWID. In the context of injection opioid use, alcohol availability alone may be insufficient to produce racially differentiated overdose vulnerability, despite well-documented inequities in alcohol outlet environments [19–24]. Instead, other structural and behavioral determinants of overdose (e.g., unstable housing, limited access to substance use disorder treatment, and criminal legal involvement) may play a more substantial role in shaping racial/ethnic differences in overdose risk among PWID [62].

We must consider several limitations of our analysis. First, the utilization of 2018 NHBS data and 2016 AOD data does not allow us to consider more recent trends in overdose and alcohol availability. Recent data have indicated that overdoses among Black Americans now outpace those among their White counterparts [15–17]. Pandemic-era increases in alcohol access through home delivery suggest that our AOD measure, which only accounts for off-premise brick-and-mortar establishments, may underestimate true alcohol availability [63], making our estimates of both AOD and its association with overdose conservative. Recent studies showing stronger post-pandemic effects of off-premise availability on alcohol-related harms further underscore this possibility [64, 65]. Nevertheless, the mechanisms highlighted in this study, particularly the role of alcohol as a potentiator of opioid overdose, remain highly relevant in the current context, where polysubstance use has become increasingly common [66]. As such, our findings underscore the continued importance of integrating alcohol-opioid co-use screening, brief behavioral interventions, and targeted naloxone distribution into overdose prevention efforts. Also, we relied on 2016 CBP data as outlet counts were suppressed in later years, and the subsequent two-year lag between exposure and outcome may have introduced bias; increases in alcohol availability after 2016 could have attenuated associations toward the null. However, AOD was highly stable between 2014 and 2016, mitigating concerns that meaningful changes during this period would have generated substantial bias.

Additionally, limitations inherent in the NHBS survey preclude more precise analyses. The survey did not ascertain alcohol co-involvement in nonfatal overdoses or alcohol and opioid co-use patterns, so we were not able to account for these factors in our analysis. The survey also does not include direct measures of engagement in harm reduction services beyond receipt of injection equipment from SSPs. Second, spatial misalignment may have also attenuated the observed associations. Using ZIP code-level AOD as a proxy for alcohol consumption assumes that PWID are purchasing and consuming alcohol near their reported residential ZIP codes, which may not reflect their actual drinking environments. Indeed, 67% of participants in this study’s sample were unhoused in the previous year, and reported residential ZIP codes may not have aligned with their actual activity spaces. However, ZIP code was assigned based on where participants reported sleeping most nights in the past year, which may partially reduce concerns about exposure misclassification. Third, CDC does not allow RDS-weighted analyses for NHBS studies to protect participant privacy and anonymity; although we adjusted for RDS seed status, unweighted analyses may yield less precise variance estimates and limit generalizability beyond the sampled PWID. Lastly, findings are not generalizable to all PWID in the US, as participants were recruited from only MSAs with high HIV/AIDS prevalences, excluding PWID residing in more rural settings. Notably, we were unable to conduct several exploratory and sensitivity analyses after losing access to NHBS data when CDC implemented a reduction in force in early 2025. These constraints underscore the need for future research leveraging additional data sources with robust measures of alcohol-related and harm reduction behaviors.

In sum, while some evidence points toward the regulation of AOD as a potential avenue to reduce overdose risk in the general population [11–13], this regulation alone may not address overdose risk among PWID specifically. However, while AOD may not be directly associated with overdose risk among PWID, it may still be related to other adverse outcomes among PWID that warrant further investigation (e.g., binge drinking, mental health distress, risky sexual behaviors). Future research should delve deeper into why AOD may not be linked to overdose among PWID as it is in the general population, exploring factors such as PWID’s intentional timing of alcohol consumption relative to injection drug use, and whether specific patterns of co-use (e.g., binge drinking while injecting) might influence overdose risk.

## Data Availability

The data used in this study are not publicly available due to Centers for Disease Control and Prevention (CDC) data use restrictions. The study team obtained access to the data through a guest researcher partnership with the CDC, which precludes us from sharing the data directly. Any data requests must be made directly to the CDC.

## Abbreviations

AOD: Alcohol outlet density
CBP: ZIP code business pattern
MSA: Metropolitan statistical area
NHBS: National HIV behavioral surveillance
ZCTA: ZIP code tabulation area
